# Origin and Genetic Diversity of *Barbatula* (Cypriniformes: Nemacheilidae) in Italy

**DOI:** 10.1002/ece3.72832

**Published:** 2026-01-05

**Authors:** Lucia Zanovello, Daniel Eisendle, Stefano Casari, Matthias Ennemoser, Hannes Grund, Gino Favrin, Simone Rossi, Andrea Modesti, Mauro Luchelli, Lukas Rüber, Andreas Meraner, Andrea Gandolfi

**Affiliations:** ^1^ Conservation Genomics Research Unit Research and Innovation Centre ‐ Fondazione Edmund Mach San Michele all'Adige Italy; ^2^ Centro Tutela Specie Acquatiche Agenzia Demanio provinciale di Bolzano Scena Italy; ^3^ Ufficio Gestione Fauna Selvatica Bolzano Italy; ^4^ Dipartimento Qualità dell'Ambiente, U.O. Biologia Ambientale e Biodiversità ARPAV Treviso Italy; ^5^ Area di Ricerca Territoriale Fondazione Lombardia per l'Ambiente Milano Italy; ^6^ Naturhistorisches Museum Bern Bern Switzerland; ^7^ Aquatic Ecology & Evolution, Institute of Ecology and Evolution University of Bern Bern Switzerland; ^8^ Amt für Jagd und Fischerei Graubünden Chur Switzerland

**Keywords:** biodiversity management, conservation, DNA barcoding, molecular taxonomy, stone loach

## Abstract

Recent morphological and molecular studies suggested the existence of several undescribed species within the genus *Barbatula*. The stone loach ( *Barbatula barbatula* ) is considered, according to the Italian Red List, as native in Northern Italy and classified as vulnerable (VU), having a limited and fragmented distribution from Lombardy to Friuli‐Venezia Giulia regions. In the present study, 248 specimens of *Barbatula* sp., collected from 17 sampling sites in Italy—spanning its entire known distribution area—and from one site in Austria, were analysed by sequencing the Cytochrome C Oxidase I (COI) and the Cytochrome B (CytB) mitochondrial regions. Sequencing results were then compared with reference samples from the literature. Three highly divergent mitochondrial lineages were observed in Italian populations, which can be associated with three different species: *Barbatula pironae* in Friuli‐Venezia Giulia, *Barbatula fluvicola* in Trentino‐Alto Adige and Lombardy, and *Barbatula* aff. *barbatula* coexisting with the latter in Lombardy. The three species, with the first having a distribution limited to the upper Adriatic area, and the other two having a wider distribution north of the Alps, should therefore be considered as different Management Units. Therefore, the integration of the Italian freshwater fish species checklist and the update of their taxonomy are strongly advised. Our data together with other available evidence suggest that the three species are likely native to Italy, and hence a revision or definition of their conservation status might be needed.

## Graphical abstract

The stone loach ( *Barbatula barbatula* Linnaeus, 1758) is considered native in Northern Italy and classified as vulnerable (VU), having a limited and fragmented distribution In the present study, 248 specimens of *Barbatula* sp., collected from sampling sites spanning its entire known distribution area in Italy, were analysed by sequencing the Cytochrome C Oxidase I (COI) and the Cytochrome B (CytB) mitochondrial regions. Three highly divergent mitochondrial lineages were observed in Italian populations, which can be associated to three different species: *Barbatula pironae* in Friuli‐Venezia Giulia, *Barbatula fluvicola* in Trentino‐Alto Adige and Lombardy, and *Barbatula* aff. *barbatula* coexisting with the latter in Lombardy.

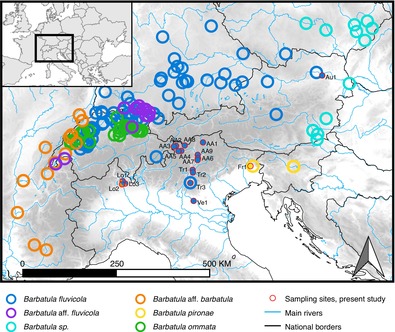

## 1 Introduction

The genus *Barbatula* Linck, 1790 comprises several species of small freshwater teleost fishes of the Eurasian region, living mainly at the bottom of fast‐flowing rivers and lacustrine habitats (Barluenga and Meyer 2005; Kottelat and Freyhof 2007). This taxon shows remarkable levels of previously unrecognized morphological, meristic, and genetic diversity, which has led to the description of numerous new species from the Asian region in recent years (e.g., Cao et al. 2012; Prokofiev 2015, 2018; Chen et al. 2019).

In Europe, similarly, in addition to the European stone loach *Barbatula barbatula* (Linnaeus, 1758), six other species are known (Fricke et al. 2025). Three of these, namely the Vardar stone loach *Barbatula vardarensis* (Karaman, 1928), the Ohrid stone loach *Barbatula sturanyi* (Steindachner, 1892) and the Zeta stone loach *Barbatula zetensis* (Sorić, 2000), are endemic to the Balkan region. The other three species are endemic to the Pyrenean area: the Languedoc stone loach *Barbatula quignardi* (Băcescu‐Meşter, 1967), the Spanish stone loach *Barbatula hispanica* (Lelek, 1987; revalidated by Denys et al. 2021) and the Leopard stone loach *Barbatula leoparda* Galliard, Detail, Preset, Keith & Denys, 2019. Several studies have suggested, however, the existence of more, yet undescribed, species among *B. barbatula* populations, identified by the presence of distinct genetic lineages (Knebelsberger et al. 2015; Behrens‐Chapuis et al. 2021; Zangl et al. 2022; Clavero et al. 2023). Overall, *B. barbatula* populations show genetic diversity patterns of isolation‐by‐distance (Barluenga and Meyer 2005; Takács et al. 2008; Deflem et al. 2022), likely due to the taxon's limited dispersal abilities (Šedivá et al. 2008). This characteristic has led some authors to suggest that each European catchment could potentially host at least one endemic stone loach species, similarly to the European minnows *Phoxinus* spp. (Denys et al. 2021). Recently, two new species have been described from Switzerland, namely *Barbatula fluvicola* Calegari et al. (2025) and *Barbatula ommata* Calegari et al. (2025). The authors also designated a neotype for the nominal species, *B. barbatula* , from the Lez River population, consequently classifying *B. quignardi* as a junior synonym of *B. barbatula* .

The presence and the native status of *Barbatula* sp. in Italy is currently controversial. Historical evidence supports the autochthony of this fish in Italy, where it has been reported as *Cobitis barbatula* since at least the mid‐1500s (Gessner 1551; and following literature). In detail, *Barbatula* sp. populations were known (mentioned as *Cobitis barbatula*) on the southern slopes of the European Alps and in the Padano‐Venetian district: in Lake Maggiore (Gessner 1551, although this data is uncertain, as detailed below), in the Adige and Fibbio rivers and in the Garda Lake (Brugnatelli 1788; Pollini 1816; de Betta 1862), in the Province of Trento (Canestrini 1866), and in the lower Friuli (Nardo 1866).

According to the International Union for the Conservation of Nature (IUCN) assessment of 2019, *B. barbatula* was classified as “Least Concern” (LC) globally, due to the occurrence of stable and abundant populations over a vast geographic range, spanning across most of Europe and including also Northern Italy (Freyhof 2019). The Italian Red List of Vertebrates, similarly, considered *B. barbatula* autochthonous in Italy but ‘Vulnerable’ (VU) to extinction based on the A3e criterion (reduction of population size caused by introduced taxa and other possible factors; Rondinini et al. 2013). This status was then confirmed in the most recent Italian Red List of Vertebrates, where the species is reported as native to Italy and VU (Rondinini et al. 2022; see also Lorenzoni et al. 2019). In the latest global IUCN assessment, however, while the conservation status of *B. barbatula* was unchanged, the updated distribution area currently shows the Italian populations as introduced and only present in the Adige River catchment (Freyhof 2024).

In the present study, we collected *Barbatula* sp. samples from four Italian basins in which populations are presently reported: the Marano and Grado Lagoon Basin (Friuli‐Venezia Giulia region: Pizzul 2020), the Adige River Catchment (Veneto: Gandolfi et al. 1991; and Trentino‐Alto Adige region: VV. AA. 2022), the Fissero Tartaro Canalbianco Basin (Veneto: VV. AA. 2023), and the Lake Maggiore Basin, within the Po Drainage (Lombardy region). This last population was reported only very recently by Puzzi et al. (2017). Although the authors outlined their finding as the first record of the taxon in the Lake Maggiore Basin, the historical distribution of the European stone loach may already have included Lake Maggiore (Gessner 1551). In fact, it should be noted that Gessner likely referred to the distribution of *Cobitis* sp. individuals, including both *Cobitis aculeata* and *C. barbatula* , in a single paragraph, as explained by Calegari et al. (2025). As no other author, to our knowledge, mentioned the historical presence of *Barbatula* sp. in this basin, this occurrence is therefore uncertain. Nonetheless, targeted surveys in small streams of the surrounding area, prompted by the discovery in 2017, led to rapidly identifying two more stone loach populations, suggesting that the lack of historical records might be due to inefficient or nonexistent research for this taxon, having limited relevance for sport fishing (Bergero et al. 2024a, 2024b). Since ichthyofauna surveys in Italy are more often fueled by fishery interests than conservation purposes, stone loach populations, as well as other species of minor fish fauna, could generally be overlooked even in other Italian basins. Very recently, the occurrence of *Barbatula* sp. individuals was reported for the first time in the Piedmont region, within an artificial water body in the high Dora Riparia River Drainage, together with exotic salmonid species typically stocked to sustain angling activities (Delmastro et al. 2023). The authors argued that the presence of this nonreproductive population is, in fact, almost certainly occasional and non‐native.

Considering the overall lack of information on genetic diversity of *Barbatula* sp. populations in Italy, as well as on their native status, and taking into account recent taxonomic revisions in the European Alpine area, the present study aimed at (i) describing, for the first time, the genetic diversity and differentiation of Italian *Barbatula* populations with mitochondrial gene markers; (ii) evaluating the existence of possible different Management/Conservation Units; and (iii) discussing the opportunity of a conservation status update for these populations if their native status is confirmed.

## 2 Methods

### 2.1 Field Methods/Sample Collection

**FIGURE 1:**
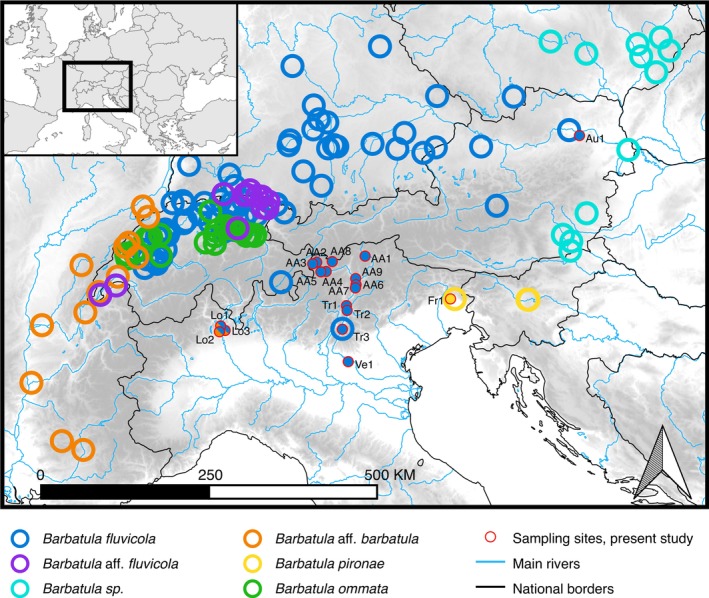
Map of the study area (Northern Italy) and surrounding Alpine region; open circles represent, in different colors, different *Barbatula* species distribution, according to table S2 in Calegari et al. (2025); sampling sites of this study are represented as labelled circles with a red circumference (AA1‐AA9: Alto Adige or Province of Bolzano; AU1: Austria; Fr1: Friuli‐Venezia Giulia; Lo1‐Lo3: Lombardy; Tr1‐Tr3: Province of Trento; Ve1: Province of Verona, Veneto), filled with colors representing the species found, as defined from present genetic data. Image created with QGIS.org (2024) QGIS Geographic Information System. Open Source Geospatial Foundation Project http://qgis.org and edited with Inkscape.

Tissue samples (fin clips) of 248 individuals of *Barbatula* sp. were collected from wild populations, covering 17 sampling sites in four Italian drainages, where this taxon is currently reported, as well as one site in the Danube drainage, Austria. Identification codes for each population (Alto Adige: AA1‐ AA9; Trentino: Tr1‐ Tr3; Veneto: Ve1; Friuli‐Venezia Giulia: Fr1; Lombardy: Lo1‐ Lo3; Austria: AU1), as well as the number of sampled individuals and geographical information on the sites, are reported in Table 1. Geographical position of the sampled populations, together with the distribution of *Barbatula* spp. in the Alpine region as proposed by Calegari et al. (2025), is shown in Figure 1.

**TABLE 1:** For each sampled population, identification code (ID Code), number of sampled individuals (N ind.), latitude, longitude, sampling date, river/drainage (B., biotope; C., creek; D., ditch; Dr., drain; L., lake; R., river), administrative region or province, and country are shown, as well as the number of individuals carrying each COI and CytB haplotype.

ID code	N ind.	Lat.	Long.	Sampling date	River/Drainage	Region/Province	Country	COI							CytB												
								h01	h02	h03	h04	h05	h06	h07	h01	h02	h03	h04	h05	h06	h07	h08	h09	h10	h11	h12	h13
AA1	27	46.806	11.504	13/10/2022	Pontelletto L./Adige	Alto Adige	Italy				27					7		13		7							
AA2	15	46.717	10.498	21/07/2022	L. Piccolo Lago del Prete/Adige	Alto Adige	Italy				15							15									
AA3	15	46.717	10.496	21/07/2022	*L. Grande* Lago del Prete/Adige	Alto Adige	Italy				15							15									
AA4	5	46.626	10.677	26/07/2022	Schienenwahl D./Adige	Alto Adige	Italy				5							4	1								
AA5	19	46.625	10.660	22/12/2022	Ontaneto di Oris B./Adige	Alto Adige	Italy				19						9	10									
AA6	15	46.417	11.306	20/10/2022	Vadena 59B D./Adige	Alto Adige	Italy				15					5		1	9								
AA7	15	46.373	11.291	20/10/2022	Vadena 59A D./Adige	Alto Adige	Italy				15					8		3	4								
AA8	9	46.736	10.841	25/07/2024	Vernago L./Adige	Alto Adige	Italy				9							9									
AA9	20	46.496	11.299	07/08/2024	Stampfl D./Adige	Alto Adige	Italy				20					12			8								
AU1	17	48.282	16.009	06/05/2022	Große Tulln/Danube		Austria		1		16											1		16			
Fr1	9	46.168	13.172	09/03/2023	Urana C./Marano and Grado Lagoon	Friuli‐Venezia Giulia	Italy	9							9												
Lo1	20	45.857	8.611	16/08/2022	Ballarate C./L. Maggiore	Lombardy	Italy			3		4	13										4		3	13	
Lo2	15	45.820	8.620	19/07/2022	Prati Magri Dr./L. Maggiore	Lombardy	Italy					4	11								4						11
Lo3	25	45.818	8.627	19/07/2022	Acquanegra C./L. Maggiore	Lombardy	Italy					8	16	1							8						17
Tr1	18	46.132	11.104	03/2023	Avisio R./Adige	Trentino	Italy				18					9		6	3								
Tr2	1	46.067	11.114	03/2023	Adige R./Adige	Trentino	Italy				1					1											
Tr3	1	45.808	11.011	03/2023	Adige R./Adige	Trentino	Italy				1					1											
Ve1	2	45.360	11.109	14/10/2024	Aosetto Dr./Fissero Tartaro Canalbianco	Veneto	Italy				2							2									
Total	248							9	1	3	178	16	40	1	9	43	9	78	25	7	12	1	4	16	3	13	28

Sampling was done by backpack electrofishing, making one pass, taking care to sample all the habitat types present. The length of the sampled section was 10–20 times the average width of the stream. All the captured specimens were measured and weighed, and a fin clip of each individual was collected with scissors and stored in pure ethanol in an alphanumerically coded tube, before being released in the same site. All sampling procedures were approved by local authorities and performed in accordance with relevant guidelines and regulations.

### 2.2 Laboratory Methods

DNA extractions were carried out with the Mag‐Bind Blood & Tissue DNA HDQ Kit (Omega BIO‐TEK, Georgia), following the manufacturer's instructions. Each extraction batch also included one negative control (water only).

For each sample, a fragment of the Cytochrome C Oxidase I (COI) gene was PCR amplified using primers LCO1490 and HCO2198 (Folmer et al. 1994), and a fragment of the Cytochrome B (CytB) gene with primers Glu‐F and Thr‐R (Zardoya and Doadrio 1998). In both cases, the PCR reaction mix included 10 μL of Promega Flexi Buffer 5X, 4 μL of MgCl₂ 25 μM, 0.5 μL of bovine serum albumin (BSA) 10 mg/mL, 1 μL of each forward and reverse primer 10 pmol/μL, 1 μL of dNTPs 10 mM each, 0.25 μL of Promega—GoTaq HS G2 5U/μL, 2 μL of template DNA and sterile H₂O up to a volume of 50 μL. The PCR program followed the Promega Taq Kit parameters, performing 35 cycles and with an annealing temperature of 50°C for COI and 56°C for CytB. Each PCR reaction included the extraction blanks and two to three PCR blanks (reaction mixture only).

PCR products were purified with Exonuclease I and Shrimp Alkaline Phosphatase (ExoSAP; Thermo Fisher Scientific, Waltham, Massachusetts, USA) according to the manufacturer's guidelines, except for DNA being diluted as 2 μL of template DNA and 2 μL ExoSAP in 3 μL of sterile water. Purified products were then sequenced with Sanger technology using the same forward and reverse primers, with the BrilliantDye Terminator Cycle Sequencing kit (v3.1; Resnova, Roma, Italy). Sequencing reaction products were run on a 3730XL Genetic Analyzer (Thermo Fisher Scientific, Massachusetts, USA).

### 2.3 Data Analysis

All sequences were aligned and quality‐checked with Geneious 2024.0.7 (http://www.geneious.com; Kearse et al. 2012). COI sequences were uploaded to the Barcode of Life Data System (BOLD; Ratnasingham and Hebert 2007). To investigate the phylogenetic relationship among our samples, Median Joining Networks (Bandelt et al. 1999) were generated with Pop ART (Leigh and Bryant 2015) using default parameters (ε = 0), first with COI and CytB sequences separately and then with the concatenated fragments.

Barcode Index Number (BIN; Ratnasingham and Hebert 2013) assignment was performed on COI sequences using BOLD Identification Engine on the Animal Library (Public) with the Rapid Species Search, which includes only reference sequences above 94% identity and a minimum overlap of 100 base pairs (bp) with query sequences. To further validate BIN assignment results, a distance‐based (“Assemble Species by Automatic Partitioning,” ASAP; Puillandre et al. 2021) and a tree‐based (“Bayesian Poisson Tree Processes” model, bPTP; Zhang et al. 2013) species delimitation methods were applied to both COI and CytB datasets. ASAP was run using the Kimura (K2P) TS/TV model and default parameters. Both COI and CytB phylogenetic input trees for the bPTP analysis were constructed using IQTree2 2.0.7 (Trifinopoulos et al. 2016) from command line, with ultrafast bootstrap approximation (UFBoot; ‐B 1000; Hoang et al. 2018), and converted to Newick format with FigTree v1.4.4 (http://tree.bio.ed.ac.uk/software/figtree/). Lastly, bPTP analyses were carried out on its webserver (https://species.h‐its.org/ptp/) with default parameters (100,000 MCMC generations, thinning = 100, burn‐in fraction = 0.1, random seed). For both datasets, results of the best partitioning based on ASAP scores and of Maximum Likelihood partitioning of bPTP are discussed here.

In addition, to compare COI haplotypes with already described genetic diversity, a reference database was created by downloading all sequences of *Barbatula* sp. available in BOLD (accessed on 12 March 2025) in FASTA format, along with their metadata in TSV format. Similarly, reference CytB sequences were retrieved from GenBank (accessed on 12 March 2025) by searching for “Barbatula[ORGN] AND cytochrome B” and downloading all data in GenBank (full) format. Sequence data were then extracted from these files with *gbmunge* (https://github.com/sdwfrost/gbmunge), while metadata information such as accession number (Acc. N.), species names, and geographical origin were obtained with a custom Python script. Both COI and CytB databases were then refined by discarding all sequences from non‐European species (except for one sequence of *Barbatula toni* in each dataset, BOLD DSBAR557‐06 and GenBank AB242162 respectively, which was used as outgroup). The resulting databases were aligned with, respectively, COI and CytB sequences from this study using the Linux package *mafft* with default parameters (Katoh et al. 2002; Katoh and Standley 2013). Each alignment was then visually checked, deleting low‐quality or very short sequences. For comparison to the reference databases, to obtain the longest possible sequences while losing as few references as possible, the original 641 bp (see Section 3) COI fragments were cut to 636 bp, removing 5 bases in the 3′ region. Similarly, the 1140 bp CytB sequences were cut to 1094 bp by removing 37 bases at the 5′ and 9 at the 3′ region. ASAP and bPTP analyses were run on each dataset including our sequences and their respective reference databases, with the same parameters as above. Finally, for both markers, the trees built with IQTree2 were used to graphically represent the putative species found within European *Barbatula* by collapsing branches with TreeViewer (Bianchini and Sánchez‐Baracaldo 2024) according to BIN (COI sequences only), ASAP, and bPTP (both markers).

## 3 Results

For all individuals included in the study, a 641 bp and a 1140 bp long sequences were obtained for COI and CytB respectively. COI sequences were submitted to BOLD under the Project BARBA (BARBA001‐24–BARBA248‐25). Both COI and CytB sequences were submitted to GenBank, corresponding to Acc. Ns PV955142–PV955389 and PV952388–PV952635, respectively.

**FIGURE 2:**
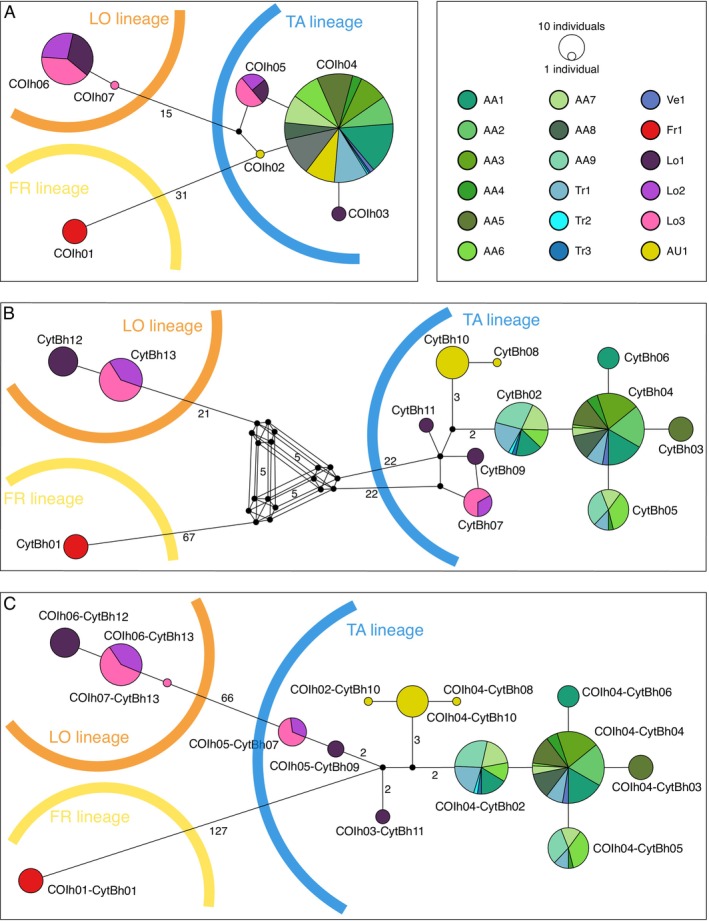
Median Joining Networks of COI (panel A), CytB (B), and concatenated sequences (C), where colors of circles represent sampled sites (black circles: haplotypes not found here). Relative dimensions of circles correspond to relative frequency of each haplotype; distances between haplotypes are not proportional to genetic distances, and the number of mutational steps is reported if more than 1. Major mtDNA (LO, TA, FR) lineages are graphically delimited by arches having the same colors used for species in Figure 1. Sampling sites are reported with the following codes: AA1, Pontelletto L.; AA2, L. Piccolo Lago del Prete; AA3, *L. Grande* Lago del Prete; AA4, Schienenwahl D.; AA5, Ontaneto di Oris B.; AA6, Vadena 59B D.; AA7, Vadena 59AD; AA8, Vernago L.; AA9, Stampfl D.; AU1, Große Tulln; Fr1, Urana C.; Lo1, Ballarate C.; Lo2, Prati Magri Dr.; Lo3, Acquanegra C.; Tr1, Avisio R.; Tr2, Adige R.; Tr3, Adige R.; Ve1, Aosetto Dr. (B., biotope; C., creek; D., ditch; Dr., drain; L., lake; R., river). Networks created with PopART and edited with Inkscape.

Seven COI haplotypes were identified in the sampled populations (COI h01‐h07), whose distribution and frequency are shown in Table 1. In detail, COI h01 was detected only in site Fr1, while the Austrian population hosted two haplotypes (COI h02 and h04). All populations of Alto Adige, Trentino, and Veneto (sites AA1‐AA9, Tr1‐Tr3, and Ve1) shared the same COI haplotype (h04), while in Lombardy (sites Lo1‐Lo3) four haplotypes were found (h03 and h05‐h07). Five out of seven haplotypes were never published before (see Table 2). Among the already known haplotypes, COI h04 sequence was identical to reference sequences from the Danubian Drainage in Austria and Germany and the Rhine Drainage in Germany (e.g., GenBank Acc. N. KM373639; Knebelsberger et al. 2015), while COI h06 corresponded to a haplotype from the Rhône Drainage in France (Acc. N. KJ553271; Geiger et al. 2014). The Median Joining Network built on COI sequences (Figure 2A) showed that haplotypes cluster into three well differentiated mitochondrial lineages: the first only found in Friuli‐Venezia Giulia Region, including only COI h01 haplotype (COI‐FR lineage); the second one observed in Alto Adige, Trentino, and Veneto with h04, in Austria with h02 and h04, and in Lombardy, with h03 and h05 (COI‐TA lineage); and the third lineage, found only in Lombardy, with the remaining two haplotypes (h06 and h07; COI‐LO lineage). Thus, populations of Lombardy included individuals from two different mitochondrial lineages (COI‐LO and COI‐TA).

**TABLE 2:** For each mtDNA marker, haplotypes detected in this study (Hapl., in bold if not published before) are reported, referred to the genetic lineage they belong to, with respective number of individuals (N ind.), BINs (for COI), and any nominal species (n.d., not determined) clustering together according to the three species delimitation analyses: BOLD BIN (COI only), ASAP, and bPTP.

Gene	Lineage	Hapl.	N ind.	BIN	BOLD BIN	ASAP	bPTP
COI	COI‐FR	**h01**	9	BOLD:AAA1243	*B. barbatula, B. vardarensis*	*B. barbatula, B. vardarensis*	n.d.
	COI‐TA	**h02**	1	BOLD:AAA1239	*B. barbatula, B*. sp.	*B. barbatula, B*. sp.	*B. barbatula, B*. sp.
		**h03**	3	BOLD:AAA1239	*B. barbatula, B*. sp.	*B. barbatula, B*. sp.	*B. barbatula, B*. sp.
		h04	178	BOLD:AAA1239	*B. barbatula, B*. sp.	*B. barbatula, B*. sp.	*B. barbatula, B*. sp.
		**h05**	16	BOLD:AAA1239	*B. barbatula, B*. sp.	*B. barbatula, B*. sp.	*B. barbatula, B*. sp.
	COI‐LO	h06	40	BOLD:ACF1394	*B. barbatula, B. quignardi*	*B. quignardi*	*B. quignardi*
		**h07**	1	BOLD:ACF1394	*B. barbatula, B. quignardi*	*B. quignardi*	n.d.
			N tot		18	24	50
CytB	CytB‐FR	**h01**	9			*B. barbatula*	*B. barbatula*
	CytB‐TA	**h02**	43			*B. barbatula*	*B. barbatula*
		**h03**	9			*B. barbatula*	*B. barbatula*
		**h04**	78			*B. barbatula*	*B. barbatula*
		**h05**	25			*B. barbatula*	*B. barbatula*
		**h06**	7			*B. barbatula*	*B. barbatula*
		**h07**	12			*B. barbatula*	*B. barbatula*
		**h08**	1			*B. barbatula*	*B. barbatula*
		**h09**	4			*B. barbatula*	*B. barbatula*
		**h10**	16			*B. barbatula*	*B. barbatula*
		**h11**	3			*B. barbatula*	*B. barbatula*
	CytB‐LO	**h12**	13			*B. barbatula, B. quignardi*	*B. barbatula, B. quignardi*
		h13	28			*B. barbatula, B. quignardi*	*B. barbatula, B. quignardi*
			N tot		n.a.	30	55

*Note:* Total number (N tot) of putative species identified in the reference databases according to the different methods is also summarized in the last row for each marker.

Thirteen CytB haplotypes (CytB h01‐h13) were identified in the present dataset, whose distribution and frequency are reported in Table 1. As for COI, a single private haplotype (CytB h01) was found in site Fr1. Individuals from site AU1 showed two haplotypes (CytB h08 and h10). Five CytB haplotypes were detected in the populations AA1‐AA9 and Tr1‐Tr3 (h02‐h06), with h06 being the rarest (found only in site AA1). The remaining five haplotypes were identified in the populations from Lombardy (h07, h09, and h11‐h13). Three of these (h09, h11, and h12) are private to the Lo1 population, while h07 and h13 are found in Lo2 and Lo3. The only previously described haplotype (CytB h13) corresponds to GenBank Acc. N. DQ025831 (unpublished), from the Saone Drainage in France (specifically, the Grosne River). The Median Joining Network of CytB sequences (Figure 2B) confirmed the existence of three major haplogroups, one limited to the only haplotype found in Fr1 (CytB‐FR lineage), the second including haplotypes h12 and h13 found in Lombardy (CytB‐LO lineage), and the third with haplotypes h02‐h11 (CytB‐TA lineage). This last haplogroup included three distinct sublineages with different geographical distributions (Figure 2B).

Lastly, the network constructed on COI and CytB concatenated sequences (Figure 2C) markedly summarized the presence of three genetic mitochondrial lineages and showed the same fine‐scaled geographical structure highlighted by the more informative CytB marker. Inside TA lineage, in fact, haplotypes from Austria, Trentino‐Alto Adige and Veneto, and Lombardy appeared to cluster as different sub‐lineages.

All species delimitation analyses performed on our samples only, namely BIN for COI sequences, and ASAP and bPTP for both markers, highlighted the existence of three mitochondrial lineages, suggesting their status of different putative species. These lineages completely corresponded, in terms of haplotypes grouping, to those shown by the Median Joining Networks.

Even after shortening the original COI and CytB fragments to create new datasets in order to include already published sequences, the number of haplotypes remained the same. In addition, all new haplotypes here defined on the entire sequence length were confirmed as not published before (i.e., not sharing 100% similarity on the retained length with any reference sequence). BIN assignment analysis of the dataset including all European COI sequences of this genus and sequences from this study identified 18 putative species overall, and three species within our new sampleset (Table 2). COI‐FR lineage haplotypes were assigned to BIN BOLD:AAA1243, which includes sequences identified as *B. barbatula* and *B. vardarensis*, while COI‐TA haplotypes corresponded to BIN BOLD:AAA1239 (identified as *B. barbatula* and *B*. sp.), and COI‐LO haplotypes to BIN BOLD:ACF1394 (including sequences identified as *B. barbatula* and *B. quignardi*). ASAP identified different numbers (24 for COI and 30 for CytB) of putative species in the overall dataset, but confirmed the separation of our sequences into three putative species for both markers (shown in Table 2). Similarly, bPTP defined even higher numbers of species overall (50 for COI and 55 for CytB), and suggested the existence of the three corresponding lineages with CytB, but provided a slightly different outcome for COI (see Table 2). In this case, COI‐LO lineage was split into two putative species, identified with *B. quignardi* (COI h06) and an undefined species (COI h07).

Tree representations of these putative species' delimitations according to the three analyses highlighted a consistent geographic pattern (Appendix 1): in fact, FR lineages (both COI and CytB) clustered together with (BIN, Appendix 1A; ASAP, Appendix 1B,D) or close to (bPTP, Appendix 1C,E) *B. barbatula* and *B. vardarensis* populations from Eastern Europe and the Balkan region; TA lineage haplotypes grouped together with *Barbatula* populations of central Europe (Austria, Germany, Switzerland); lastly, LO lineage showed the highest similarity with *B. barbatula* and *B. quignardi* populations from France, and less consistently, Poland, Russia, and Switzerland. This pattern proved to be congruent when analysing both COI and CytB sequences, despite some differences due to heterogeneity of available references for the two gene markers (e.g., no CytB sequences of *B. leoparda* nor *B. hispanica* were found in GenBank).

## 4 Discussion

This study provided the first characterization of genetic diversity in *Barbatula* sp. populations in Italy, based on two mitochondrial sequence markers. Overall, within the sampled populations, seven COI and 13 CytB haplotypes of *Barbatula* sp. were identified, most of which were never described before (five COI and 12 CytB haplotypes). Both markers highlighted the existence of three well‐differentiated mitochondrial lineages, possibly representing as many different species, whose occurrence was only partially described in Italy. While targets for conservation actions, that is, Management/Conservation Units, are often identified with populations and not species, taxonomy is at the base of listing adopted in official guidelines for conservation (e.g., in IUCN Red Lists and/or in the national and local normative framework). Therefore, the association of these lineages to described species is a necessary step in order to allow their formal recognition and protection (e.g., Mace 2004). In fact, all species delimitation analyses performed here support the split of Italian samples into at least three distinct species, corresponding to the three major haplogroups. Moreover, the bPTP analysis on the COI dataset including also published references suggested the split of LO lineage into two distinct species, one of which is represented by a single individual and only differing by a single mutation (see COI h07 in Figure 2). This outcome, different from the other analyses results, is likely explained by the unbalanced number of individuals sampled per species included in the analysis, as cautioned by the authors of the software bPTP (Zhang et al. 2013).

The finding of multiple *Barbatula* species contrasts with previous relevant assessments in the geographical area under investigation (Rondinini et al. 2022; Freyhof 2024), but is in agreement with available scientific literature in neighboring regions. In fact, numerous recent barcoding studies highlighted cases of unrecognized diversity, and possibly the occurrence of new undescribed species, in stone loach populations of surrounding European areas (e.g., Germany: Knebelsberger et al. 2015; Behrens‐Chapuis et al. 2021; Austria: Zangl et al. 2022; Spain: Clavero et al. 2023; Switzerland: Calegari et al. 2025). Contextualization of our sequences within the frame of European *Barbatula* sp. genetic diversity allowed confirming these results, as well as to provide relevant insights on the phylogenetic relationships between Italian stone loaches and the other European populations/species.

### 4.1 Mitochondrial Lineages and Phylogeographic Relationships

The FR mitochondrial lineage was found in the only sampling site of Friuli‐Venezia Giulia (Fr1) and is represented by only one COI and one CytB haplotype, both newly described and fixed and private in the population. The presence of *Barbatula* individuals was reported as *B. barbatula* in a few sites and different basins in this region between 2009 and 2017, but seems to be rare and sporadic (Pizzul 2020). Thus, we were able to collect specimens in this site only. The FR lineage appears to be the most genetically differentiated of the three Italian ones, as shown by the haplotype networks (Figure 2). This putative species is closely related to other *Barbatula* populations living east of the Alps in the Balkan region, including both *B. barbatula* and *B. vardarensis* nominal species. This genetic cluster is part of the “Danubian” clade (DC/Vc) according to Šedivá et al. (2008) based on CytB analysis (e.g., Acc. Ns EF562694, EF562702) and corresponds to BIN BOLD:AAA1243 (also found in Austria: Zangl et al. 2022) based on COI marker. The status of distinct species was proposed for a *Barbatula* population of this area as far back as the Nineteenth Century when the Italian naturalist Nardo reported the finding of a rare stone loach, endemic to the lower Friuli‐Venezia Giulia and locally called *patrou*, mentioning it as a plausible new species to be described under the name of [*Cobitis*] *pironae* (Nardo 1866). The species was however never formally described and is currently considered a synonym of *B. barbatula* (Bánki et al. 2025). According to Calegari et al. (2025), however, *B. pironae* would represent a valid species, currently awaiting a formal description. These authors collected samples of this putative species from the Isonzo/Soča Basin (Italy) and the Danubian Drainage (Slovenia). Unfortunately, no common references were found between their study and the present work. However, our Fr1 sampling site is located particularly close to their sampling site in the Isonzo/Soča Basin (6.5 km linear “crow‐fly” distance), although the two belong to different basins thus having no direct freshwater continuity. Considering that the putative species′ distribution according to Calegari et al. (2025) encompasses different basins, our samples belonging to the same species seem the most parsimonious scenario.

Both COI and CytB haplotypes of TA lineage appeared to be closely related to other haplotypes from *Barbatula* sp. populations of the Danubian Drainage (in Austria, Germany, and Switzerland), possibly representing the same species (BIN BOLD:AAA1239, Behrens‐Chapuis et al. 2021; Zangl et al. 2022). Reference CytB haplotypes nested within our TA haplogroup included Acc. Ns EF562634‐36, which were identified as part of the *B. barbatula* “Western clade” (WC/I) in the Danube catchment (Vils and Isar rivers) by Šedivá et al. (2008). As haplotype COI h04 corresponded to Acc. N. KM373639, which is one of the reference sequences for the newly described species *Barbatula fluvicola* (Western European lineage W1) according to Calegari et al. (2025), and the whole lineage clustered together with other reference sequences for *B. fluvicola* in their study (e.g., Acc. Ns ON097749, ON097489), TA lineage is nested within this newly described species. *B. fluvicola* was, in fact, reported in the upper and middle Rhine Drainage (Switzerland and Germany), in the upper Danube Drainage (Germany and Austria), in the Inn Drainage (Austria and Switzerland), and in the Po Drainage in Italy (Calegari et al. 2025). In particular, our Austrian site AU1, included in this study as an outgroup with respect to Italian samples, is situated along a small inflow of the Danube River (Große Tulln) very close to the Danube River and downstream of the Traisen inflow, which was a sampling site of Calegari et al.'s study. In addition, their southernmost sampling site in the Inn Drainage is located, in Switzerland, about 60 and 115 km (linear “crow‐fly” distance) from our Adige AA3 and Lake Maggiore Lo1 sites, respectively. However, the minimum linear distance between Inn and Adige, and between Inn and Po (including Lake Maggiore), or respective inflows, is definitely shorter, as these basins are bordering. Furthermore, and more importantly, Calegari et al. (2025) reported the presence of this species in a single site in the Po Drainage; however, the geographic coordinates reported by the same authors for this site correspond almost exactly to those of our Adige Tr3 site (about 500 m linear distance). Therefore, assuming that the coordinates provided by Calegari et al. are correct, we can conclude that all our data confirm the presence of *B. fluvicola* in the Adige Basin as the only *Barbatula* species. The Fissero Tartaro Canalbianco Basin, despite currently being a separate basin with respect to the Adige Basin, has been in connection with Adige, Tosigono, and likely other rivers, in historical times (Biscaccia 1865). The presence of *B. fluvicola* in this basin (site Ve1) is therefore not surprising. The same species is found in the Lake Maggiore inflows as well, but sympatric with LO lineage (detailed below). Nonetheless, all COI and CytB haplotypes of TA lineage, but COI h04, represent genetic diversity here described for the first time. This data thus represents relevant information in order to understand the plausibility of the native status for this species in Italy (discussed below).

LO lineage consisted of two COI and two CytB haplotypes, with one of each new and the other already published. According to BIN and ASAP species delimitations, this lineage represents a putative species including other, mostly French, populations assigned, depending on authors, to *B. barbatula* (e.g., Acc. N. DQ025831, unpublished) or *B. quignardi* (e.g., Geiger et al. 2014). No reference sequences from Šedivá et al. (2008), whose work focused on the Danube Drainage, were found grouping together with CytB‐LO haplotypes. Comparing the reference sequences in common between this study and the one by Calegari et al. (2025), LO lineage should correspond to Western European lineage W2 (e.g., KJ553271, exactly matching our COI h06, is a reference sequence for this lineage). The taxon corresponding to Western European lineage W2 was previously referred to as *B. quignardi* (e.g., Geiger et al. 2014) but, according to Calegari et al., should now be considered *B. barbatula* . Furthermore, the same authors reported evidence for the existence, within this lineage, of two morphospecies, namely the redescribed *B. barbatula* and the yet undescribed *B*. aff. *barbatula*. In addition to morphological differences, these two morphospecies show a distinct geographical distribution, as far as known from currently available data. LO lineage appears genetically closer to the morphospecies *B*. aff. *barbatula* Western European lineage W2, whose distribution includes the Rhône and Rhine drainages (rivers Doubs, Allaine, Le Bied, Areuse, Broye, Venoge, and Lake Geneva). This result is coherent with the geographical proximity of our sampling sites in Lake Maggiore to *B*. aff. *barbatula* sites with respect to *B. barbatula* distribution (Figure 1).

### 4.2 Native Status of *Barbatula* in Italy

Although only *B. barbatula* is currently recognized in Italy (Rondinini et al. 2022), genetic evidence from this study suggests the presence of three different *Barbatula* species south of the Alps: *B. fluvicola*, *B*. aff. *barbatula*, and *B. pironae* (Calegari et al. 2025). A possible and conservative explanation is that the presence of one or more species of those found in the present study, especially in the case of the Lombardy sampling sites where two different species are found in sympatry, could be the effect of human‐mediated translocations. Similar natural co‐occurrence of congeneric species is generally possible if ecological and reproductive barriers are established (e.g., Gause 2019). To our knowledge, given the currently accepted taxonomy, *Barbus* represents the only genus with two species having sympatric or parapatric populations in the present study area. This could, on the other hand, be interpreted as a possible effect of inaccurate and insufficiently defined taxonomy for several genera. This limitation, together with a lack of knowledge on genetic diversity, is a general issue for the Italian freshwater fish fauna, especially the ones of limited interest for fishery and angling (e.g., Zanovello et al. 2025). In the case of *Barbatula*, known for centuries in Italy and since then considered as a single species, a partial systematic revision of the genus, with the formal description of new species or the recognition of new species yet undescribed (Calegari et al. 2025), allowed in fact to identify unexpected species diversity south of the Alps.

Clarifying the native (or exotic) status of these new putative species and populations is fundamental in order to define proper management measures and, if needed, conservation practices. The definition of the native status of a species in a specific area is, however, not trivial, and becomes increasingly complicated along with the direct and indirect effects of human activities. While numerous scientific papers have been published on criteria to assess potential risks posed by exotic species (e.g., Roy et al. 2014; Nentwig et al. 2016), only a few guidelines for the identification of native/exotic status have been proposed so far (e.g., Webb 1985). In detail, Webb (1985) proposed eight different criteria, some of which are relevant and can be appropriately evaluated in the case of *Barbatula* species in Italy.

#### 4.2.1 Historical Evidence

Records of *Barbatula* species, known at the time as [*Cobitis*] *barbatula*, in Italy date back to the mid‐1500s (Gessner 1551) and have been consistently reported for most areas here investigated in the successive literature (e.g., Brugnatelli 1788; de Betta 1862; Canestrini 1866; Nardo 1866; Scotti 1898). The only exception is the population of Lake Maggiore in Lombardy, which was not recorded between 1556 (Gessner 1551) and 2017 (Puzzi et al. 2017). Thus, the possibility of one or both species present within this population being the result of recent introductions at this site cannot be immediately ruled out. However, as previous surveys of wild fish fauna in this area have focused almost exclusively on river stretches suitable for sport fishing target species (Bergero et al. 2024b), and considering this genus' limited body size, elusive behavioral ecology, and lack of angling interest, it is also possible that these stone loach populations were completely overlooked until their rediscovery by Puzzi et al. (2017). Their report of *Barbatula* sp. individuals at site Lo3, in fact, rapidly led to finding two more populations in the area (Lo1 and Lo2), when small streams potentially suitable for this taxon were actively searched (Bergero et al. 2024a, 2024b). Additionally, historical literature provides support for stone loach presence in Italian culture, such as the existence of local dialectal names (e.g., Italy: *fondola*, Gessner 1551; Verona: *strega*, Brugnatelli 1788; de Betta 1862; Friuli‐Venezia Giulia: *forapière*, Pirona 1854; lower Friuli: *patrou*, Nardo 1866), and the use of this fish as food (see also below; Raimondi 1630; Configliachi and Brugnatelli 1808; de Betta 1862). As in any other cases, in the absence of fossil records, historical documents alone cannot constitute definitive proof against the hypothesis of translocations operated in previous times.

#### 4.2.2 Level of Anthropization of the Site

A high level of anthropogenic impact on the water bodies where *Barbatula* was newly described could suggest the possibility that these populations are non‐native. In general, especially in the case of non‐salmonid fish fauna, stocking activities involve the reproduction and growth of individuals in seminatural environments, then recaptured and translocated to the recipient water body. This practice could lead to the unintended translocation of other fish species (hitchhiking; Thaulow et al. 2014). Furthermore, in habitats highly impacted by exotic top predator fish (e.g., *Salmo* or *Salvelinus*), prey fish are commonly introduced to sustain the feeding requirements of the former, thus representing another case of exotic taxa associated with extensively altered habitats (Corral‐Lou et al. 2019). In the case of *Barbatula* in Italy, the new population found in Piedmont by Delmastro et al. (2023) occurs in a highly anthropized water body, where an entirely exotic fish community is also present. Thus, the occurrence of *Barbatula* sp. in this region through stocking is highly likely.

#### 4.2.3 Geographical Distribution of Species

According to this criterion, the geographic location of newly found species needs to be evaluated in relation to the known distribution of the same species. In fact, the occurrence in areas continuous or discontinuous with respect to known native distribution needs to be explainable by plausible phylogeographic scenarios. The finding of different congeneric species in a single ichthyogeographic district (Padano‐Venetian district, see Bianco 1990), as is the case here discussed, should therefore be compatible with natural multiple colonization scenarios in order to consider a native status for the observed species. Although the mountain system of the Alps is perceived as a single and cohesive barrier to the migration and diffusion of taxa, transalpine territories are part of a very extensive and diverse region, spanning different basins and major drainages. Consequently, it is not unreasonable to think that different access routes to the Italian peninsula and different refuge areas, during the glacial periods, may have existed for primary species (sensu Bănărescu 1990) of a single genus. A known example in the Padano‐Venetian district is the genus *Barbus*, represented by two endemic species, namely *Barbus caninus* and *B. plebejus*, which are more closely related to other transalpine species rather than to each other (respectively, to *B. balcanicus* and to *B. macedonicus* ; Levin et al. 2019; Rossi et al. 2021). These two taxa thus most likely derived from independent colonization events. Moreover, a similar scenario of highly divergent *Barbatula* lineages coexisting in the same river system following different colonization events has been previously hypothesized by Šedivá et al. (2008) for populations of the Danube Basin. According to the authors, these cold‐water adapted species could have diverged during a warmer period (possibly in the Miocene), surviving in isolated mountain refuges in the Alps and Carpathians, and have subsequently recolonized lowland basins leading to secondary contacts of the diverged lineages. Similarly, Calegari et al. (2025) described an overlapping distribution of genetically supported different species in the central Alps (see also Figure 1). In particular, two species having a non‐sister group relationship, namely *B. fluvicola* and *B. ommata*, shared part of their geographic range in the upper Rhine, suggesting allopatric speciation followed by range expansion and secondary contact (Calegari et al. 2025).

In our case, we attributed FR lineage (Friuli‐Venezia Giulia) to *B. pironae* (available name). Although this species is still undescribed, and defining its expected distribution area is therefore inherently complicated, the sampling site where this lineage was observed is certainly coherent with the originally reported location of *B. pironae* (i.e., “basso Friuli”, lower Friuli; Nardo 1866). For both TA and LO lineage, as discussed in detail above, our sampling sites are located in correspondence (i.e., Adige Basin sites) or close to (i.e., Lake Maggiore) known sites where the two species are respectively found: Adige and high Inn rivers for *B. fluvicola*, and Rhône Drainage for *B*. aff. *barbatula*.

A striking example of multiple colonization events across the entire Alpine arc is represented by *Cottus gobio* (Šlechtová et al. 2004), whose populations in northern Italy most likely originated from independent range expansion events: in the upper Po Basin from the Roya River (France; see also Splendiani et al. 2020); in the western Po Drainage from the Rhône River; and in the Brembo and Adda rivers from the Rhine and Danube drainages (Switzerland and Austria). Finally, the authors suggested a further colonization through the Alps from the Isonzo/Soča Basin (Italy) to the upper Sava Basin (Slovenia). On the other hand, single colonization events across the Alps have been hypothesized for different species. For instance, Kotlìk and Berrebi (2002) and Tsigenopoulos et al. (2002) suggested the occurrence of gene flow between the Isonzo/Soča and Sava basins for *Barbus* populations. Meraner et al. (2007) reported the presence, with high frequency, of Danubian haplotypes of brown trout (*Salmo* sp.) in the eastern South Tyrolean (Alto Adige) rivers, and hypothesized that an ancestral drainage shortcut could have formed in the area of the Rienz/Rienza‐Drave watershed between the Adriatic and Black Sea drainage basin, leading to a natural introgression from the Drave River. An identical scenario has been proposed for *Thymallus thymallus* in the same area (Meraner et al. 2014). However, in the case of *Thymallus*, the authors suggested a historical stocking scenario as the most likely, based on Approximated Bayesian Computation model testing. Lastly, an unidirectional corridor for migration from the Po watershed to the upper Durance River catchment would have permitted the colonization of empty habitats by *Salmo* sp. populations during the early Holocene (Splendiani et al. 2020).

#### 4.2.4 Supposed Means of Introduction and Ease of Known Naturalization Elsewhere

Historically, stone loaches constituted an appreciated food source in Italy (e.g., Raimondi 1630; Configliachi and Brugnatelli 1808; de Betta 1862), and as such could potentially have been translocated. Stocking for sport fishing and culinary purposes has, in fact, been documented for other fish species. For example, *Salvelinus* sp., which was a refined food for the nobility, has been stocked since 1500 in high‐mountain lakes of the Trentino–Alto Adige region (Pechlaner 1984; Machino 1999). Historical Italian literature mentions a translocation event of *Barbatula* sp. from Germany to Sweden occurred about a century earlier (Gera 1850; also reported by Lundberg and Svanberg 2010, and by Norén et al. 2018). In this case, similarly to that of *Salvelinus*, the translocation would have been carried out at the orders of the local sovereign (King Fredrik I), and populations of stone loaches would have been introduced in Lake Mälaren, possibly fish ponds of the royal palace (Norén et al. 2018). However, Norén et al. questioned the plausibility of this translocation event, and the exotic origin of these populations is therefore uncertain. More recently, Clavero et al. (2023) reported the occurrence of an undescribed *Barbatula* species (likely corresponding to *Barbatula* sp. Western lineage W4 in Calegari et al. 2025) in Catalonia with unknown complete native range distribution. The authors speculated that these populations were originally introduced in the Segre Basin, which was then the source of secondary introductions in other areas. Since COI haplotypes found in Catalonia are private to this region, we suggest that interpreting these populations as native could be an alternative and possibly more conservative hypothesis.

To further add to the Italian populations' origin, historical literature reports that *Barbatula* individuals were only eaten by the poorest people, due to their modest size (de Betta 1862). Therefore, the plausibility of long‐range translocations of stone loaches being carried out across the Alps by the poorest part of the population seems unlikely. In fact, to the best of our knowledge, no historical records exist on translocation events of stone loaches to or from Italy. In addition, no explicit mentions of the use of *Barbatula* sp. individuals as live bait are available in the historical literature, to our knowledge.

#### 4.2.5 Genetic Diversity

In this study, the Italian populations are characterized by the presence of a clear majority of previously undescribed haplotypes, for both COI and CytB markers. Thus, if an exotic origin is hypothesized for these populations, it is not possible to identify the source sites. Moreover, most of these haplotypes are also private for different basins, suggesting multiple origins for them, as discussed above. The most striking example is offered by the Lombardy sampling sites, where site‐specific genetic diversity was observed: in both Lo1, on the one hand, and Lo2 and Lo3, on the other, two species were found, both represented by only private CytB haplotypes. Considering an exotic origin for all differently geographically distributed haplotypes requires as many independent translocation events to have taken place.

The occurrence in sympatry of two different mitochondrial lineages, associated to as many species, namely *B. fluvicola* and *B*. aff. *barbatula*, in the Lake Maggiore Basin, could suggest a possible hybridization scenario. Such a case was reported by Calegari et al. (2025) in two streams close to Lake Zürich, in a secondary contact zone between *B. fluvicola* and *B. ommata*. The authors could hypothesize, based on morphological data, the presence of hybrid specimens, showing intermediate phenotype and color pattern between the two species. Since in the present work no morphological data were collected, nor nuclear genetic data were analyzed, the actual presence of hybrid individuals is not supported and will need further investigation.

## 5 Conclusions

In conclusion, extant Italian populations of *Barbatula* sp. include at least three genetically distinct mitochondrial lineages, possibly corresponding to three different species, here identified as *B. pironae* (available name), *B. fluvicola*, and *B*. aff. *barbatula* (yet undescribed) (Calegari et al. 2025). One of these, namely *B. fluvicola*, also presents a genetic and geographic substructure within the investigated sampleset, allowing the identification of three mitochondrial subclusters. The first subcluster corresponds to the Austrian population AU1, the second to the Alto Adige, Trentino, and Veneto populations (AA1‐AA9, Tr1‐Tr3, and Ve1), and the last to the Lombardy populations (Lo1‐Lo3). All the different criteria we considered in order to evaluate the native or exotic status of each of these lineages in Italy—encompassing historical evidence, sites' anthropization, species' geographical range, means and modes of introduction and ease of naturalization, and genetic diversity—are compatible with a scenario of natural presence, through multiple past colonization events, of the identified species. An alternative hypothesis of historical multiple translocations cannot be conclusively ruled out. Nonetheless, recent translocations hypothesis could not in any case explain the observed genetic distribution of *Barbatula* in Italy, given the above considerations.

Therefore, an integration of the Italian freshwater fish species checklist, encompassing an update of their taxonomy and a formal description of the yet undescribed species, is urgently needed. Moreover, assuming with a precautionary approach that these lineages are native in Italy, considering the “diagnosability” criteria based on mitochondrial differentiation and their respective geographic distribution, the described populations should theoretically be treated as separate Management or Conservation Units. Taking into account the finding of several private haplotypes and the limited distribution of different lineages in Italy, in particular the single population of *B. pironae* in Friuli‐Venezia Giulia, they should be at least the object of in situ protection actions. The limited and localized geographical areas in which these populations are nowadays found should therefore be protected with active conservation measures aimed at reducing potential risk factors. *Ex situ* protection actions should be limited to cases where high threat levels are defined, following monitoring programs and ecological investigations. If the native status can be confirmed in the future, an increase of the present occupancy area should be promoted in order to minimize the risk of local population extinction. In addition, the investigation of nuclear genetic diversity together with the collection of morphometric and meristic data of Italian stone loaches will support a more comprehensive interpretation of their evolutionary history and population structure.

## Author Contributions

**Lucia Zanovello:** data curation (lead), formal analysis (lead), visualization (equal), writing – original draft (lead), writing – review and editing (equal). **Daniel Eisendle:** conceptualization (equal), funding acquisition (equal), resources (equal), writing – review and editing (equal). **Stefano Casari:** investigation (lead), writing – review and editing (equal). **Matthias Ennemoser:** resources (equal), writing – review and editing (equal). **Hannes Grund:** resources (equal), writing – review and editing (equal). **Gino Favrin:** resources (equal), writing – review and editing (equal). **Simone Rossi:** resources (equal), writing – review and editing (equal). **Andrea Modesti:** resources (equal), writing – review and editing (equal). **Mauro Luchelli:** resources (equal), writing – review and editing (equal). **Lukas Rüber:** conceptualization (supporting), writing – review and editing (equal). **Andreas Meraner:** conceptualization (equal), funding acquisition (equal), project administration (supporting), supervision (equal), writing – review and editing (equal). **Andrea Gandolfi:** conceptualization (equal), funding acquisition (equal), project administration (lead), supervision (equal), visualization (equal), writing – original draft (supporting), writing – review and editing (equal).

## Conflicts of Interest

The authors declare no conflicts of interest.

## Data Availability

The complete dataset of *Barbatula* sp. COI sequences produced and analysed in this study has been uploaded in BOLD (Project name: BARBA; BARBA001‐24–BARBA248‐25). Both COI and CytB datasets were also submitted to GenBank (Acc. Ns PV955142–PV955389 and PV952388–PV952635, respectively).

## Appendix 1

COI (A, B, C) and CytB (D, E) trees built with IQTree2 on the datasets including reference sequences from BOLD (COI) and GenBank (CytB), with branches collapsed according to BIN (A), ASAP (B and D), and bPTP (C and E) clustering. BINs (A) or numbers (B–E) are used to identify each putative species suggested by each analysis; respective nominal species name and, when available, country of origin for reference samples (ISO‐coded; https://www.ncbi.nlm.nih.gov/books/NBK7249/) are also reported; haplotypes observed in the present study are shown in bold, and their respective mtDNA (LO, TA, FR) lineages are highlighted using the same colors used for relevant species in Figures 1 and 2.
