# Correction to “Origin and Genetic Diversity of *Barbatula* (Cypriniformes: Nemacheilidae) in Italy”

**DOI:** 10.1002/ece3.73051

**Published:** 2026-02-04

Zanovello, L., D. Eisendle, S. Casari, et al. 2026. “Origin and Genetic Diversity of *Barbatula* (Cypriniformes: Nemacheilidae) in Italy.” *Ecology and Evolution* 16, no. 1: e72832. https://doi.org/10.1002/ece3.72832.

Appendix 1 of the published article should have included a two‐page figure comprising five sub‐figures (labeled A to E). However, the published version of this Appendix only contains a single‐page figure with three sub‐figures (labeled A to C). This has been amended in order to grant the reader complete access to the data presented in the paper.

The updated Appendix 1 is shown below.

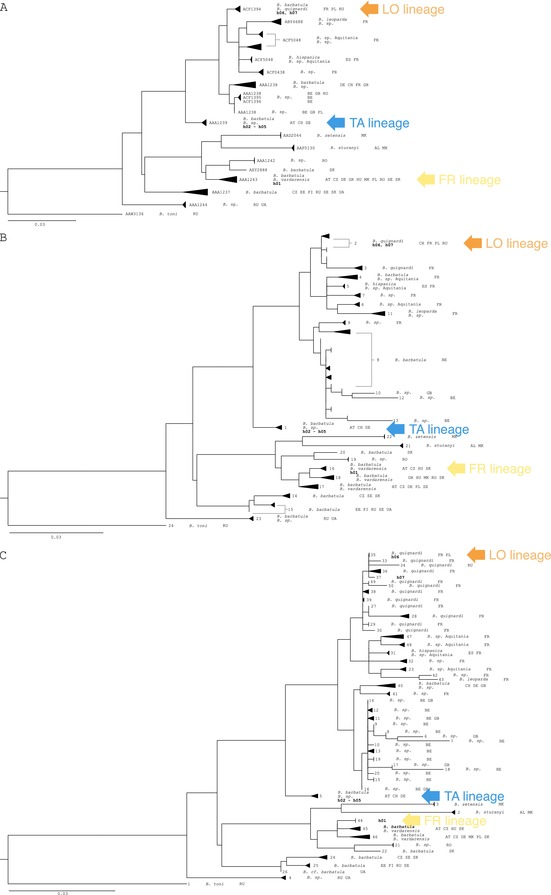

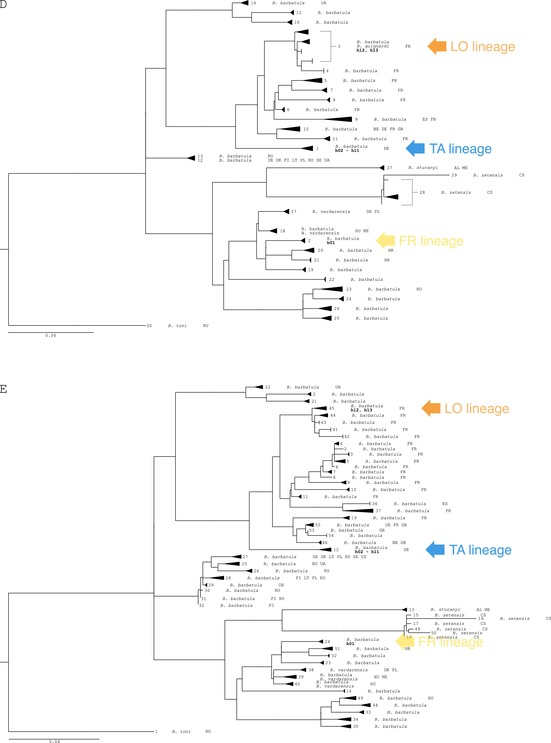

We apologize for this error.

